# Frailty Transitions in Older Persons With Lung Function Impairment: A Population-Based Study

**DOI:** 10.1093/gerona/glac202

**Published:** 2022-10-13

**Authors:** Sara R A Wijnant, Elizabeth Benz, Annemarie I Luik, Fernando Rivadeneira, Trudy Voortman, Guy G Brusselle, Lies Lahousse

**Affiliations:** Department of Respiratory Medicine, Ghent University Hospital, Ghent, Belgium; Department of Epidemiology, Erasmus MC―University Medical Center Rotterdam, Rotterdam, the Netherlands; Department of Epidemiology, Erasmus MC―University Medical Center Rotterdam, Rotterdam, the Netherlands; Department of Internal Medicine, Erasmus MC―University Medical Center Rotterdam, Rotterdam, the Netherlands; Department of Epidemiology, Erasmus MC―University Medical Center Rotterdam, Rotterdam, the Netherlands; Department of Internal Medicine, Erasmus MC―University Medical Center Rotterdam, Rotterdam, the Netherlands; Department of Epidemiology, Erasmus MC―University Medical Center Rotterdam, Rotterdam, the Netherlands; Division of Human Nutrition and Health, Wageningen University and Research, Wageningen, the Netherlands; Department of Respiratory Medicine, Ghent University Hospital, Ghent, Belgium; Department of Epidemiology, Erasmus MC―University Medical Center Rotterdam, Rotterdam, the Netherlands; Department of Epidemiology, Erasmus MC―University Medical Center Rotterdam, Rotterdam, the Netherlands; Department of Bioanalysis, Faculty of Pharmaceutical Sciences, Ghent University, Ghent, Belgium

**Keywords:** Epidemiology, Frailty, Pulmonary, Resilience

## Abstract

**Background:**

The aging population and its burden on health care systems warrant early detection of patients at risk of functional decline and mortality. We aimed to assess frailty transitions and its accuracy for mortality prediction in participants with impaired spirometry (Preserved Ratio Impaired Spirometry [PRISm] or chronic obstructive pulmonary disease [COPD]).

**Methods:**

In participants from the population-based Rotterdam Study (mean age 69.1 ± 8.9 years), we examined whether PRISm (forced expiratory volume in 1 second [FEV_1_]/forced vital capacity [FVC] ≥ 70% and FEV_1_ < 80%) or COPD (FEV_1_/FVC < 70%) affected frailty transitions (progression/recovery between frailty states [robust, prefrailty, and frailty], lost to follow-up, or death) using age-, sex- and smoking state-adjusted multinomial regression models yielding odds ratios (OR). Second, we assessed the diagnostic accuracy of frailty score for predicting mortality in participants with COPD using c-statistics.

**Results:**

Compared to participants with normal spirometry, participants with PRISm were more likely to transit from robust (OR 2.2 [1.2–4.2], *p* < .05) or prefrailty (OR 2.6 [1.3–5.5], *p* < .01) toward frailty. Participants with PRISm (OR 0.4 [0.2–0.8], *p* < .05) and COPD (OR 0.6 [0.4–1.0], NS) were less likely to recover from their frail state, and were more likely to progress from any frailty state toward death (OR between 1.1 and 2.8, *p* < .01). Accuracy for predicting mortality in participants with COPD significantly improved when adding frailty score to age, sex, and smoking status (90.5 [82.3–89.8] vs 77.9 [67.2–88.6], *p* < .05).

**Conclusion:**

Participants with PRISm or COPD more often developed frailty with poor reversibility. Assessing physical frailty improved risk stratification for participants with impaired spirometry for predicting increased life years.

With the aging population, more people suffer from chronic illnesses, increasing the importance of early detection of the most vulnerable in order to improve quality of life and prevent a high burden on health care systems (1). Physical frailty, as defined by the Fried frailty phenotype, is a clinical syndrome in which a progressive, cumulative decline in the reserve capacity of multiple physiological systems elicits a state of increased vulnerability to stressor events and susceptibility to adverse outcomes (2). Distinction of frail older people from those that are robust is essential in an aging population, particularly in those with several chronic conditions, because frailty changes their therapeutic approach and prognosis (3). In addition, the possibility to delay frailty on the one hand, and poor reversibility of frailty on the other hand, warrants health systems to shift toward preventive primary care to timely detect prefrailty (4).

It is becoming increasingly evident that frailty plays a role in worse health outcomes of patients with chronic obstructive pulmonary disease (COPD), and conversely that COPD may increase the risk of frailty (3,5,6). Participants with COPD and participants with frailty share risk factors such as aging, smoking, and common pathophysiological mechanisms of chronic inflammation, immune system dysfunction, and impaired neuroendocrine regulation (5). Longitudinal studies observed that having COPD or respiratory impairment (airflow limitation or restrictive-pattern) was associated with a higher risk of transferring from robust to prefrail/frail (5,7,8). Participants with COPD were also less likely to recover from frailty compared to participants without COPD (5,9). Longitudinal studies on frailty in participants with Preserved Ratio Impaired Spirometry (PRISm) are lacking to date, despite the increasing awareness for PRISm as an important spirometric phenotype associated with mortality (10).

Assessing frailty transitions in participants with lung function impairment might help to identify those persons with COPD at high risk of functional decline and mortality. Therefore, our first aim was to investigate how lung function impairment (PRISm or COPD) affects frailty transitions in a large population-based cohort study. Second, we aimed to examine mortality in participants with lung function impairment and frailty combined. Third, within participants with impaired spirometry, we aimed to examine the accuracy for mortality prediction of the physical frailty score.

## Method

### Study Design and Population

This study was conducted within the Rotterdam Study, a population-based cohort study that started in 1990 in Rotterdam, the Netherlands, comprising almost 15 000 participants aged ≥45 years. The Rotterdam Study aims to assess the occurrence of, and risk factors for, chronic diseases in the older (11). Every 4–5 years, participants undergo a home interview and clinical examinations at the research center. Implementation of spirometry started in 2009. Participants were included in the study if an interpretable spirometry was completed at one (baseline cross-sectional analysis between 2009 and 2014) or 2 study visits (longitudinal analysis with follow-up between 2014 and 2016, Supplementary Figure 1).

The Rotterdam Study has been approved by the Medical Ethics Committee of the Erasmus MC (registration number MEC 02.1015) and by the Dutch Ministry of Health, Welfare and Sport (Population Screening Act WBO, license number 1071272-159521-PG). All participants provided written informed consent to participate in the study and to have their information obtained from treating physicians.

### COPD Assessment and Classification

Prebronchodilator spirometry was performed by trained paramedical personnel using a Master Screen PFT Pro (Care Fusion, Houten, the Netherlands) according to the ATS/ERS guidelines (12). Postbronchodilator spirometry and total lung capacity were not available in participants of the Rotterdam Study. For diffusion capacity of the lung, the transfer factor using carbon monoxide was used corrected for hemoglobin (DLCO [mmol/min/kPA]) (13). Predicted forced expiratory volume in 1 second (FEV_1_) and forced vital capacity (FVC) values were calculated using Global Lung Initiative reference equations taking age, sex, height, and ethnicity into account (14). Normal spirometry (controls; FEV_1_/FVC ≥ 70% and an FEV_1_ ≥ 80%), PRISm (FEV_1_/FVC ≥ 70% and FEV_1_ < 80%), and COPD (FEV_1_/FVC < 70%) were distinguished. Severity of obstruction was determined according to the Global Initiative for Chronic Obstructive Lung Disease (GOLD) criteria: mild COPD (GOLD1, FEV_1_ ≥ 80%) and moderate to very severe COPD (GOLD2–4, FEV_1_ < 80%).

### Frailty Assessment

We determined frailty using the physical definition of frailty by Fried et al., which is the most widely used and validated instrument in frailty research (2,15,16). The definition and assessment of the Fried frailty phenotype within the Rotterdam Study have been extensively described previously (17). Frailty was defined as meeting 3 or more of the 5 established criteria evaluating (a) weight loss, (b) low physical activity (18), (c) slow gait velocity (19), (d) reduced grip strength, and (e) self-reported exhaustion (20,21) (Supplementary Table 1). These criteria are in line with the criteria used to define frailty by Fried et al., except that weight loss was not self-reported over the previous year, but derived from directly measured weight at each center visit and covered a time span of multiple years. Only participants who had a sufficient number of criteria to confirm or to exclude frailty were included (ie, at least 3 concordant positive or negative criteria evaluated). Participants fulfilling 1 or 2 criteria were defined as prefrail. Participants fulfilling no criteria were defined as robust.

### Covariables

Smoking status (never, former, and current) and pack-years (years smoked multiplied by a daily number of smoked cigarettes divided by 20) were assessed by interview. Information on medication use was obtained from interview and from pharmacy records. Venous blood samples for the determination of levels of serum glucose, cholesterol, hemoglobin, and DNA were obtained using an automated enzymatic method (22).

We calculated a composite comorbidity score for each participant by summing one point for each fulfillment of the following nine comorbidity criteria. Hypertension was defined as a systolic blood pressure ≥ 140 mmHg, a diastolic blood pressure ≥ 90 mmHg, or the use of blood pressure-lowering drugs (23). Clinical diagnosis of heart failure, stroke, coronary heart disease, diabetes, and cancer were based on or included active follow-up using the medical records of the participants (22,23). Coronary heart disease includes myocardial infarction, coronary artery bypass grafting, and percutaneous coronary intervention (23). Diabetes was defined as a fasting plasma glucose level ≥ 7 mmol/L, a nonfasting plasma glucose level ≥ 11.1 mmol/L, or the use of glucose-lowering medication applying World Health Organization (WHO) criteria (24). Kidney disease was defined according to the National Kidney Foundation guidelines as having an estimated glomerular filtration rate (eGFR) <60 mL/min/1.73 m). The eGFR was obtained based on the formula as provided by the Chronic Kidney Disease Epidemiology Collaboration (25). Osteoporosis was defined using femoral neck and lumbar spine bone mineral density measured by Dual-energy x-ray absorptiometry applying WHO criteria (26). Anemia was defined according to the WHO guidelines <120 g/L hemoglobin for women and <130 g/L hemoglobin for men (27).

### Statistical Analysis

We compared baseline characteristics between robust, prefrail, and frail participants using the Student’s *t* test, Mann–Whitney *U* test, and chi-square test, as appropriate, and calculated incidence rates (IR) for frailty per 1 000 person-years in different lung function categories (normal spirometry, PRISm, and COPD) after excluding prevalent cases.

Age, sex, and smoking status-adjusted multinomial logistic regression models yielding odds ratios (OR) evaluated whether lung function affected frailty progression (ie, the likelihood of transitioning from a robust toward a prefrail/frail state compared with maintenance of a robust state, or the likelihood of transitioning from any frailty state toward death or lost to follow-up compared with active follow-up) and frailty recovery (ie, the likelihood of transitioning from a prefrail/frail state toward a robust state compared with maintenance of a prefrail/frail state).

We calculated mortality rate per 1 000 person-years in different lung function categories (normal spirometry, PRISm, or COPD) and frailty states (robust, prefrail, or frail), and compared age- and sex-adjusted Cox proportional hazard ratios for mortality in participants with or without the combination of frailty and impaired spirometry. Schoenfeld residuals were checked to test whether the assumptions for Cox models were satisfied.

To assess whether adding physical frailty score to a model with age, sex, and smoking status improved the prediction of mortality in participants with impaired spirometry, we used receiver operating characteristic curves with the areas under the curve (AUCs). The nonparametric method described by DeLong et al. was used to compare differences in AUCs across different combinations of tests (28). All statistical analyses were performed using R version 3.6.

## Results

### Study Population

**Figure 1.**
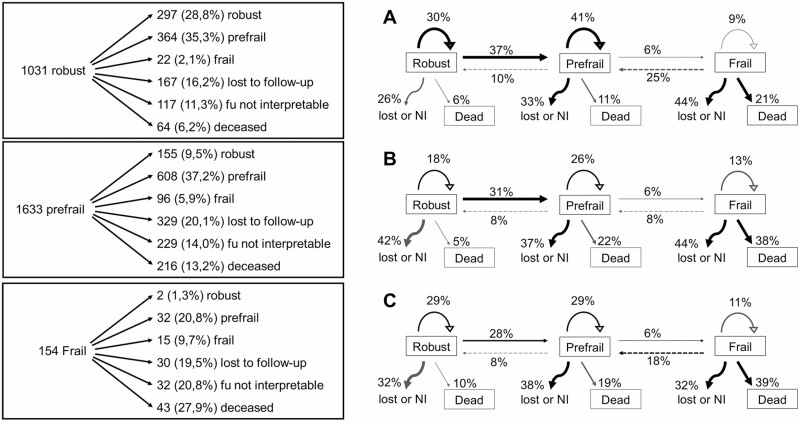
Transition of frailty status in participants with interpretable frailty and spirometry measures at baseline that were invited for a follow-up visit (*n* = 2 818). Transition of frailty status in (**A**) participants with normal spirometry, (**B**) participants with PRISm, and (**C**) participants with COPD at baseline. Dead = no follow-up visit and date of death before last visit date of round 6 of the Rotterdam Study, Lost = lost to follow-up (no follow-up visit in a survivor), not interpretable = recorded follow-up visit in survivor but either spirometry or frailty assessment is not interpretable. NI = not interpretable. Transitions between robust and frail were omitted from the right panel of the figure. PRISm = Preserved Radio Impaired Spirometry; COPD = chronic obstructive pulmonary disease.

At baseline, the physical frailty status could be determined for 5 442 participants with interpretable spirometry (mean age 69.1 ± 8.9 years, 55.8% female sex): 183 (3.4%) out of 5 442 participants met the criteria for being frail, and 2 656 (48.8%) for prefrail (Table 1). A comparison of the clinical characteristics of the 16.6% participants with COPD and 7.0% participants with PRISm to those 76.4% at baseline with normal spirometry is presented in Supplementary Table 2. Of 5 442 included participants at baseline, 2 818 participants were invited for reexamination between 2014 and 2016 (follow-up). Of those, 323 had died, 526 were lost to follow-up, and 378 had not interpretable spirometry or frailty assessments at the follow-up visit (Supplementary Figure 1). Thus, 1 591 of reinvited participants had both interpretable spirometry and their frailty status determined twice (mean age 73.8 ± 4.9 years, 55.0% female sex) and were included in the longitudinal analysis: 49 (3.1%) out of these 1 591 participants met the criteria for frailty, and 859 (54.0%) participants met the criteria for prefrailty at baseline. Comparison of clinical characteristics of participants with and without reexamination is presented in Supplementary Table 3. The median time between baseline and follow-up examination was 4.5 years (interquartile range [IQR] 4.3–4.9). The number of participants who transitioned toward a different frailty category between baseline and follow-up after a median of 4.5 years (IQR 4.3–4.9) is shown in Figure 1.

**Table 1.** Baseline Characteristics of Participants That Meet the Criteria for Frailty or Prefrailty (*n* = 5 442)

	Robust	Prefrail	p*	Frail	p*
	*N* = 2 603	*N* = 2 656		*N* = 183	
Age (years)	66.3 (7.6)	**71.3 (9.0)**	**<.001**	**77.7 (8.5)**	**<.001**
Female sex (%)	1 353 (52.0%)	**1** **553 (58.5%)**	**<.001**	**132 (72.1)**	**<.001**
BMI (kg/m²)	27.3 (4.1)	**27.8 (4.5)**	**<.001**	27.4 (5.2)	.751
Current smoking (%)	296 (11.4%)	345 (13.0%)	.080	23 (12.6%)	.710
Pack-years (years)	4.5 (0.0–21.0)	**6.0 (0.0–25.0)**	**.006**	2.4 (0.0–27.0)	.777
Total cholesterol	5.6 (1.1)	**5.4 (1.1)**	**<.001**	**5.0 (1.0)**	**<.001**
Glucose	5.7 (1.1)	**5.9 (1.4)**	**<.001**	**5.9 (1.7)**	**.015**
Hemoglobin	8.9 (0.7)	**8.7 (0.8)**	**<.001**	**8.4 (0.8)**	**<.001**
White blood cell count	7.0 (2.0)	**7.2 (2.0)**	**.003**	**7.4 (2.1)**	**.022**
Serum creatinine	80.4 (17.4)	80.8 (24.2)	.476	81.4 (36.4)	.503
Slow gait speed (%)	0 (0.0%)	**21 (1.7)**	**<.001**	**28 (30.4)**	**<.001**
Weight loss (%)	0 (0.0%)	**868 (33.6)**	**<.001**	**136 (74.7%)**	**<.001**
Low physical activity (%)	0 (0.0%)	**435 (18.9)**	**<.001**	**95 (64.6)**	**<.001**
Low grip strength (%)	0 (0.0%)	**1** **406 (53.4)**	**<.001**	**166 (92.2)**	**<.001**
Exhaustion (%)	0 (0.0%)	**614 (23.3)**	**<.001**	**140 (76.5)**	**<.001**
PRISm (%)	150 (5.8%)	**214 (8.1%)**	**.001**	17 (9.3%)	.075
COPD (%)	379 (14.6%)	**471 (17.7%)**	**.002**	**53 (29.0%)**	**<.001**
FEV_1_ % predicted	99.2 (17.3)	**96.0 (19.0)**	**<.001**	**88.6 (22.7)**	**<.001**
FVC % predicted	99.9 (14.9)	**96.7 (16.1)**	**<.001**	**91.6 (17.7)**	**<.001**
FEV_1_/FVC	76.5 (7.0)	**75.9 (7.9)**	**.009**	**73.4 (11.4)**	**<.001**

*Notes*: BMI = body mass index; COPD = chronic obstructive pulmonary disease; FEV_1_ = forced expiratory volume in one second; FVC = forced vital capacity; PRISm = Preserved Ratio Impaired Spirometry. Bold values indicate statistically significant results (*p* < 0.05).

*p values are comparing characteristics of prefrail or frail participants, respectively, with robust participants.

### Association Between Lung Function and Frailty Progression

Between baseline and follow-up, 118 out of the 1 542 nonfrail participants at baseline transitioned toward incident frailty (cumulative incidence 7.7%, IR 16.6 per 1 000 person-years). In these robust and prefrail participants, the incidence of frailty was higher in participants with PRISm at baseline (cumulative incidence 14.3%, IR 31.0 per 1 000 person-years, IR ratio [IRR] 2.1) and in participants with COPD at baseline (cumulative incidence 9.0%, IR 19.6 per 1 000 person-years, IRR 1.3), than in participants with normal spirometry at baseline (cumulative incidence 7.0%, IR 15.1 per 1 000 person-years). Participants with incident frailty had lower lung function values at baseline, even after adjustment for age, sex, body mass index (BMI), and current smoking (*p* < .001 for FEV_1_ % predicted and *p* = .002 for FVC % predicted).

In multinomial logistic regression analyses, progression toward a frail state was more likely in older participants, female participants, and in participants with lower FEV_1_ % predicted, lower FVC % predicted or PRISm at baseline, whereas the progression of frailty status toward death or lost to follow-up/not interpretable follow-up was more likely in older, male, or current smoking participants, and in participants with lower FEV_1_ % predicted, lower FVC % predicted, PRISm, or COPD at baseline (Table 2).

**Table 2.** Factors Associated With Frailty Progression

	Incidence Frailty			Progression Toward Lost to Follow-up/Not Interpretable/Death					
	Robust to Prefrail	Prefrail to Frail	Robust to Frail	Robust to Lost/NI	Prefrail to Lost/NI	Frail to Lost/NI	Robust to Death	Prefrail to Death	Frail to Death
	*N* = 364	*N* = 96	*N* = 22	*N* = 284	*N* = 558	*N* = 62	*N* = 64	*N* = 216	*N* = 43
Model A									
Age	**1.1 (1.1–1.1)**	**1.1 (1.0–1.1)**	**1.2 (1.1–1.2)**	**1.2 (1.2–1.3)**	**1.1 (1.1–1.1)**	1.0 (1.0-1.1)	**1.4 (1.3–1.4)**	**1.2 (1.2–1.3)**	**1.1 (1.1–1.2)**
Female sex	**1.3 (1.1–1.7)**	**1.7 (1.1–2.5)**	**2.2 (1.5–3.4)**	**1.6 (1.2–2.0)**	1.2 (1.0–1.5)	0.7 (0.5–1.1)	0.9 (0.6–1.2)	**0.6 (0.5–0.9)**	**0.4 (0.2–0.6)**
Past vs never smoking	1.1 (0.9–1.5)	1.2 (0.8–1.9)	1.4 (0.9–2.2)	0.9 (0.7–1.2)	0.8 (0.7–1.0)	0.7 (0.4–1.0)	1.2 (0.8–1.8)	1.1 (0.8–1.5)	0.9 (0.5–1.4)
Current vs never smoking	0.8 (0.5–1.2)	0.8 (0.3–1.8)	0.6 (0.2–1.5)	1.1 (0.7–1.8)	1.4 (1.0–2.1)	1.9 (0.8–4.5)	**3.0 (1.7–5.1)**	**3.7 (2.3–6.1)**	**4.9 (2.0–12.2)**
FEV_1_ % predicted*	**0.9 (0.9–1.0)**	**0.8 (0.7–0.9)**	**0.8 (0.7–0.8)**	**0.8 (0.8–0.9)**	**0.9 (0.9–0.9)**	**1.1 (1.0–1.2)**	**0.8 (0.7–0.8)**	**0.8 (0.8–0.9)**	1.0 (0.9–1.1)
Model B									
FVC % predicted*	**0.9 (0.8–1.0)**	**0.8 (0.7–0.9)**	**0.7 (0.6–0.8)**	**0.8 (0.7–0.8)**	**0.9 (0.8–1.0)**	**1.0 (1.0–1.3)**	**0.7 (0.7–0.8)**	**0.8 (0.7–0.9)**	1.0 (0.9–1.2)
Model C									
PRISm vs NL spirometry	1.2 (0.7–2.0)	**2.3 (1.2–4.2)**	**2.6 (1.3–5.5)**	**2.1 (1.2–3.6)**	**1.8 (1.2–2.6)**	0.8 (0.4–1.5)	**2.8 (1.5–5.2)**	**2.4 (1.5–3.9)**	**1.1 (0.5–2.1)**
COPD vs NL spirometry	0.9 (0.6–1.2)	1.6 (1.0–2.6)	1.4 (0.8–2.4)	1.4 (1.0–1.9)	**1.5 (1.2–2.0)**	1.0 (0.6–1.6)	**1.7 (1.1–2.5)**	**1.9 (1.3–2.7)**	**1.2 (0.7–2.0)**

*Notes*: Data are presented as OR (95% CI) with *p* values from multinomial logistic regression analyses. Model A is fully depicted in the table. Models B and C are similar to model A with the exception that models B and C include FVC % predicted and spirometric groups, respectively, instead of FEV_1_ % predicted as a covariate. *OR per 10% change in FEV_1_ % predicted or FVC % predicted are depicted. COPD = chronic obstructive pulmonary disease; FEV_1_ = forced expiratory volume in 1 second; FVC = forced vital capacity; NL = normal; PRISm = Preserved Ratio Impaired Spirometry; OR = odds ratio; CI =confidence interval; NI = not interpretable.

Bold values indicate statistically significant results with *p* < 0.05. Bold and underlined values indicate statistically significant results with *p* < 0.01.

### Association Between Lung Function and Frailty Recovery

The large majority of 183 frail participants at baseline were lost to follow-up/not interpretable follow-up (40.3%) or died (27.9%) before the follow-up visit. Out of 49 participants with frailty at baseline also interpretably assessed at follow-up, 34 transitioned toward robust or prefrail. Out of 908 participants with frailty or prefrailty at baseline also interpretably assessed at follow-up, 157 transitioned toward robust (CI 17.3%, IR 37.3 per 1 000 person-years). Participants with reversibility of frailty or prefrailty toward robust were younger at baseline and had higher FEV_1_, and FVC % predicted values (Supplementary Table 4).

In multinomial logistic regression analyses, recovery of frailty toward a less severe frailty state was more likely in younger participants, female participants, and in participants with higher FEV_1_ % or FVC % predicted values at baseline (Table 3). The prevalence of PRISm or COPD in those still assessable impeded recovery of frailty (Table 3).

**Table 3.** Factors Associated With Frailty Recovery

	Prefrail to Robust	Frail to Prefrail
	*N* = 155	*N* = 32
Model A		
Age	**0.9 (0.9–0.9)**	**0.9 (0.9–1.0)**
Female sex	**0.8 (0.6–1.0)**	**0.6 (0.4–0.9)**
Past vs never smoking	0.9 (0.7–1.1)	0.8 (0.5–1.2)
Current vs never smoking	1.3 (0.8–2.0)	1.3 (0.6–3.1)
FEV_1_ % predicted*	**1.1 (1.0–1.2)**	**1.2 (1.1–1.3)**
Model B		
FVC % predicted*	**1.1 (1.0–1.2)**	**1.3 (1.1–1.4)**
Model C		
PRISm vs NL spirometry	0.9 (0.5–1.5)	**0.4 (0.2–0.8)**
COPD vs NL spirometry	1.1 (0.8–1.6)	0.6 (0.4–1.0)

*Notes*: Data are presented as OR (95% CI) with *p* values from multinomial logistic regression analyses. Model A is fully depicted in the table. Models B and C are similar to model A with the exception that models B and C include FVC % predicted and spirometric groups, respectively, instead of FEV_1_ % predicted as a covariate. *OR per 10% change in FEV_1_ % predicted or FVC % predicted are depicted. COPD = chronic obstructive pulmonary disease; FEV_1_ = forced expiratory volume in 1 second; FVC = forced vital capacity, NL = normal; PRISm = Preserved Ratio Impaired Spirometry; OR = odds ratio; CI = confidence interval. Two cases that transited from frail to robust were omitted from the table.

Bold values indicate statistically significant results with *p* < 0.05. Bold and underlined values indicate statistically significant results with *p* < 0.01.

### Association of Lung Function Impairment and Frailty With Mortality

**Figure 2.**
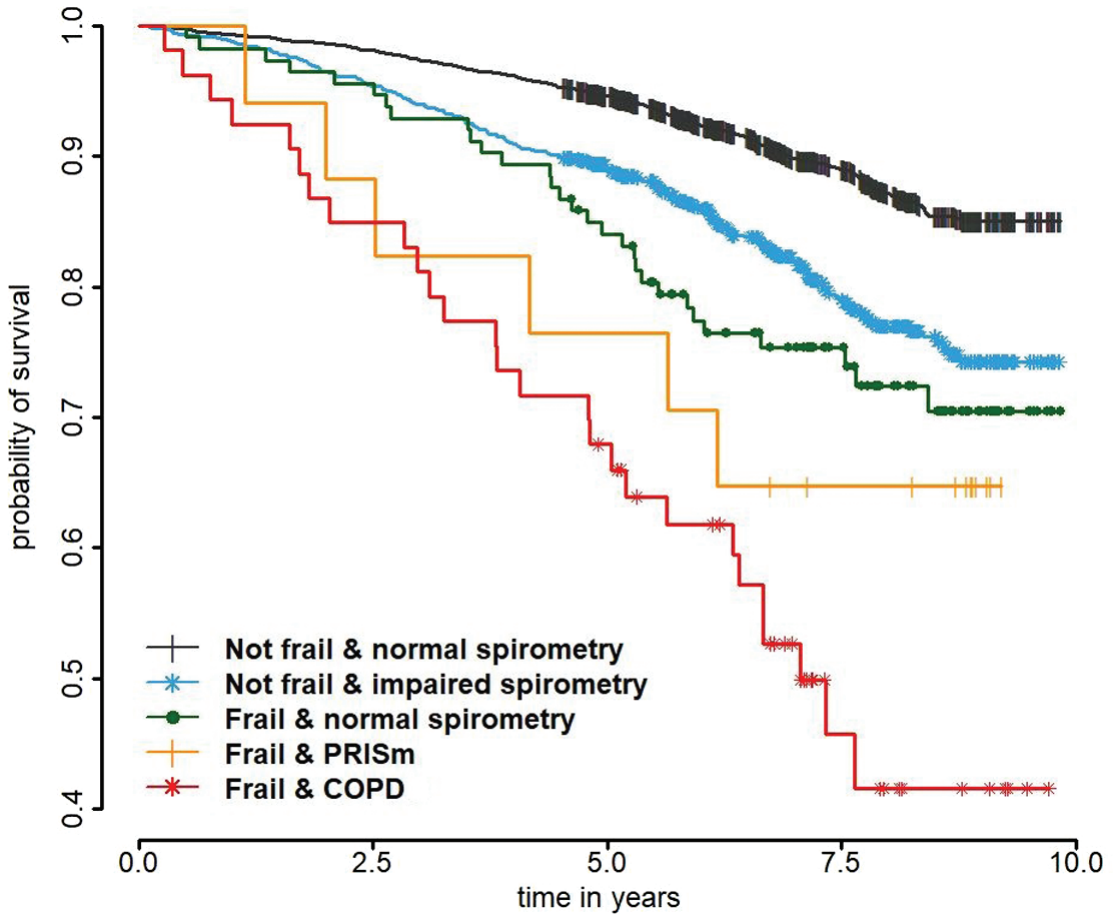
Kaplan–Meier plot for survival in participants with frailty measures at baseline (*n* = 5 414). COPD = chronic obstructive pulmonary disease, PRISm = Preserved Ratio Impaired Spirometry.

Within a maximum follow-up of 9.8 years from baseline, 678 out of 5 414 participants with informed consent for follow-up died: 157 (6.1%) robust participants, 458 (17.3%) prefrail participants, and 63 (34.4%) frail participants (Supplementary Table 5). Participants with lung function impairment (PRISm or COPD) and frailty combined had unadjusted mortality rates worse than participants with normal spirometry and frailty (Figure 2). Compared to frail participants with normal spirometry, additional COPD was an independent significant predictor of all-cause mortality in age- and sex-adjusted Cox proportional hazard regression analyses (Supplementary Table 5). Survival in nonfrail participants (robust or prefrail) with COPD GOLD1 was similar to survival in nonfrail participants with normal spirometry (Supplementary Figure 2, panel C).

### Predicting Mortality in COPD

Accuracy for predicting 1-year (90.5 [82.3–98.8]) and 3-year mortality (78.9 [73.7–84.2]) significantly improved when adding frailty score to a model with age, sex, and smoking status (Supplementary Table 6). Accuracy for predicting 5-year (83.6 [79.9–87.2]) and 9-year mortality (86.5 [83.8–89.3]) significantly improved when adding frailty score, lung function, and comorbidities to a model with age, sex, and smoking status. However, accuracy for predicting 5-year and 9-year mortality did not improve when adding frailty score to a model with age, sex, smoking status, lung function, and comorbidities.

## Discussion

Results of this large population-based cohort study advocate a complementary role for impaired spirometry and physical frailty as a tool to identify vulnerable older participants and COPD patients with an increased mortality risk. First, in the longitudinal analyses of the total population, a lower FEV_1_ and FVC % predicted were associated with the progression of frailty status, whereas a higher FEV_1_ and FVC were associated with a recovery of frailty. Second, frailty and impaired spirometry together posed a significantly higher risk of mortality compared to either frailty or impaired spirometry alone. Third, diagnostic accuracy for predicting 1-year and 3-year mortality in participants with COPD improved when adding frailty score to a model with age, sex, and smoking status. Moreover, frailty was able to distinguish in mild COPD those with increased mortality risk from those with a mortality risk equal to participants with normal spirometry.

Participants from this study with lower FEV_1_ and FVC % predicted values at baseline were more likely to become frail after a median of 4.5 years of follow-up, whereas participants with higher FEV_1_ and FVC % predicted values at baseline were more likely to recover from frailty. Similarly, participants with PRISm or COPD at baseline were more likely to transit from a robust state toward prefrailty or frailty, as well as toward death. We confirmed that the combination of frailty and COPD results in a more than the additive risk of mortality (8), and moreover add that participants with PRISm at baseline are particularly vulnerable to develop frailty as well (29). We previously observed that participants with persistent PRISm had higher BMI and more frequent chronic heart failure, and both have been recently shown to be independent risk factors for frailty in older patients with multimorbidity (10,30). Results of this article affirm the clinical importance of PRISm, a relatively prevalent spirometric phenotype that has been understudied in the past, and indicate that FEV_1_ reductions are associated with frailty even if these are aligned with reductions in FVC.

Given a large number of people with mild COPD worldwide (31), and the importance of identifying patients with poor prognosis, the Fried Frailty tool may provide a useful tool for risk stratification. Early identification of prefrailty in COPD allows for preventive and therapeutic interventions, where a key goal is to prevent the occurrence of incident frailty, clinical deterioration, and death. Moreover, in prefrail persons, it is key to further distinguish those with a transition toward frailty or poor survival versus those with the recovery of their frail state and better survival, 2 groups almost equally large in number. Herein, the results of this study advocate a role for lung function in the risk stratification of these older participants. Whether prevention of frailty progression in persons with impaired spirometry is feasible is a topic of debate and should be further investigated.

Combining the Fried Frailty phenotype with age, sex, and smoking status improved accuracy for predicting mortality in participants with COPD. Different factors were associated with either short-term (1-year and 3-year) or longer-term (5-year and 9-year) mortality. More specifically, frailty score, but not lung function, improved accuracy for predicting 1-year or 3-year mortality, advocating a role for frailty as a screening instrument for predicting short-term mortality. In contrast, the lung function or comorbidity score resulted in a greater improvement of accuracy for predicting 5-year and 9-year mortality than the frailty score. A poor prognosis in patients with COPD has been predicted using different frailty assessment tools or modifications of the BODE index (3,4,6,32,33). However, not all frailty indices provide an equally robust association with mortality as the Fried Frailty phenotype, and the phenotype appears to identify other vulnerable participants than the BODE index (7,34).

This study has several strengths. First, a large number of participants with both spirometry and frailty measures were recruited from the general population, ensuring generalizability to an older target population. Second, with a follow-up duration of up until 10 years and repeated measurements of both spirometry and frailty, this study adds to previous publications in the field with shorter follow-up times. With this, we improve risk stratification by means of frailty and lung function impairment combined. Third, although PRISm is a phenotype previously often neglected in research, we investigated frailty not only in participants with COPD, but also in participants with PRISm. Fourth, longitudinal analyses of frailty are sparse, especially in combination with longitudinal spirometry measurements, as done in this study.

Also, limitations must be taken into account. Despite the population-based cohort setting, we used data from the fifth and sixth rounds of the Rotterdam Study. Results of this study, especially those of the longitudinal part with repeated measures, may be susceptible to a healthy survivor bias. We addressed this by including participants with loss to follow-up and death in our longitudinal multinomial logistic regression analyses: those with frailty or impaired spirometry at baseline were more likely to be lost at follow-up, were more likely to have a not interpretable spirometry/frailty assessment at follow-up and were more likely to die in between the 2 study visits (Figure 1, Table 2, Supplementary Figure 2). These numbers imply better health in those that were reassessed, meaning that we likely drew inferences in a more healthy selection of the general population, typically resulting in a dilution of effect estimates. Our results argue in favor of identifying both respiratory impairment and frailty as early as possible, even if airflow limitation is still mild (COPD GOLD 1) or even if FVC reductions align FEV_1_ reductions (PRISm), although the feasibility of such screening and cost-effectiveness of linked actions to increase resilience should be further investigated. Second, prebronchodilator spirometry was used to identify participants with COPD, which might be considered a limitation, although it has proven useful and in line with other population-based cohort studies (35). Third, we measured frailty by means of the most widely used Fried Frailty phenotype, a purely physical dimensional instrument. While showing similar or even better accuracy in predicting adverse events as compared to other frailty instruments, the Fried Frailty phenotype may be challenging to implement in clinical practice, considering the need for physical space, time, and tools (4).

To conclude, participants with PRISm or COPD frequently developed frailty with poor reversibility and poor prognosis. Results of this study indicate that spirometry in combination with the physical frailty assessment, improved risk stratification for participants with impaired spirometry for predicting increased life years. Especially in the large group of people with still mild COPD worldwide, adequate risk stratification by means of frailty assessment may help clinicians in identifying patients with better or worse prognosis.

## Supplementary Material

glac202_suppl_Supplementary_Material

## Notes

Take home message: Lung function and physical frailty state have a complementary role in identifying vulnerable older participants and chronic obstructive pulmonary disease patients with an increased risk for mortality.

## References

[1] Dent E , Morley JE, Cruz-Jentoft AJ, et al Physical frailty: ICFSR international clinical practice guidelines for identification and management. J Nutr Health Aging. 2019;23:771–787. doi:10.1007/s12603-019-1273-z 31641726 PMC6800406

[2] Fried LP , Tangen CM, Walston J, et al Frailty in older adults: evidence for a phenotype. J Gerontol A Biol Sci Med Sci. 2001;56:M146–M156. doi:10.1093/gerona/56.3.m146 11253156

[3] Lee SY , Nyunt MSZ, Gao Q, et al Co-occurrence of physical frailty and COPD and association with disability and mortality: Singapore longitudinal ageing study. CHEST Elsevier. 2022;161:1225–1238. doi:10.1016/j.chest.2021.12.633 34914976

[4] Si H , Jin Y, Qiao X, Tian X, Liu X, Wang C. Predictive performance of 7 frailty instruments for short-term disability, falls and hospitalization among Chinese community-dwelling older adults: A prospective cohort study. Int J Nurs Stud. 2021;117:103875. doi:10.1016/j.ijnurstu.2021.103875 33621721

[5] Marengoni A , Vetrano DL, Manes-Gravina E, Bernabei R, Onder G, Palmer K. The relationship between COPD and frailty. Chest. 2018;154:21–40. doi:10.1016/j.chest.2018.02.014 29477493

[6] Hanlon P , Lewsey J, Quint JK, et al Frailty in COPD: an analysis of prevalence and clinical impact using UK Biobank. BMJ Open Respir Res. 2022;9:e001314. doi:10.1136/bmjresp-2022-001314 PMC9255399 35787523

[7] Lee L , Auyeung T, Leung J, Kwok T, Woo J. Transitions in frailty states among community-living older adults and their associated factors. J Am Med Dir Assoc. 2014;15:281–286. doi:10.1016/j.jamda.2013.12.002 24534517

[8] Vaz Fragoso C , Enright P, McAvay G, Van Ness P, Gill T. Frailty and respiratory impairment in older persons. Am J Med. 2012;125:79–86. doi:10.1016/j.amjmed.2011.06.024 22195532 PMC3246194

[9] Pollack LR , Litwack‐Harrison S, Cawthon PM, et al Patterns and predictors of frailty transitions in older men: the Osteoporotic Fractures in Men Study. J Am Geriatr Soc. 2017;65:2473–2479. doi:10.1111/jgs.15003 28873220 PMC5681371

[10] Wijnant SRA , Roos ED, Kavousi M, et al Trajectory and mortality of preserved ratio impaired spirometry: the Rotterdam Study. Eur Respir J. 2020;55:1901217. doi:10.1183/13993003.01217-2019 31601717

[11] Ikram MA , Brusselle GGO, Murad SD, et al The Rotterdam Study: 2018 update on objectives, design and main results. Eur J Epidemiol. 2017;32:807–850. doi:10.1007/s10654-017-0321-4 29064009 PMC5662692

[12] Celli BR , MacNee W, Agusti A, et al Standards for the diagnosis and treatment of patients with COPD: a summary of the ATS/ERS position paper. Eur Respir J. 2004;23:932–946. doi:10.1183/09031936.04.00014304 15219010

[13] Graham BL , Brusasco V, Burgos F, et al 2017 ERS/ATS standards for single-breath carbon monoxide uptake in the lung. Eur Respir J. 2017;49:1600016. doi:10.1183/13993003.00016-2016 28049168

[14] Quanjer PH , Stanojevic S, Cole TJ, et al Multi-ethnic reference values for spirometry for the 3-95-yr age range: the global lung function 2012 equations. Eur Respir J. 2012;40:1324–1343. doi:10.1183/09031936.00080312 22743675 PMC3786581

[15] de Vries NM , Staal JB, van Ravensberg CD, Hobbelen JSM, Olde Rikkert MGM, Nijhuis-van der Sanden MWG. Outcome instruments to measure frailty: a systematic review. Ageing Res Rev. 2011;10:104–114. doi:10.1016/j.arr.2010.09.001 20850567

[16] Bouillon K , Kivimaki M, Hamer M, et al Measures of frailty in population-based studies: an overview. BMC Geriatr. 2013;13:64. doi:10.1186/1471-2318-13-64 23786540 PMC3710231

[17] Lahousse L , Maes B, Ziere G, et al Adverse outcomes of frailty in the elderly: the Rotterdam Study. Eur J Epidemiol. 2014;29:419–427. doi:10.1007/s10654-014-9924-1 24935872

[18] Caspersen CJ , Bloemberg BP, Saris WH, Merritt RK, Kromhout D. The prevalence of selected physical activities and their relation with coronary heart disease risk factors in elderly men: the Zutphen Study, 1985. Am J Epidemiol. 1991;133:1078–1092. doi:10.1093/oxfordjournals.aje.a115821 2035512

[19] Verlinden VJA , van der Geest JN, Hoogendam YY, Hofman A, Breteler MMB, Ikram MA. Gait patterns in a community-dwelling population aged 50 years and older. Gait Posture. 2013;37:500–505. doi:10.1016/j.gaitpost.2012.09.005 23018028

[20] Radloff LS . The CES-D scale: a self-report depression scale for research in the general population. Appl Psychol Meas. 1977;1:385–401. doi:10.1177/014662167700100306

[21] Beekman AT , Deeg DJ, Van Limbeek J, Braam AW, De Vries MZ, Van Tilburg W. Criterion validity of the Center for Epidemiologic Studies Depression scale (CES-D): results from a community-based sample of older subjects in the Netherlands. Psychol Med. 1997;27:231–235. doi:10.1017/s0033291796003510 9122304

[22] Ikram MA , Brusselle G, Ghanbari M, et al Objectives, design and main findings until 2020 from the Rotterdam Study. Eur J Epidemiol. 2020;35:483–517. doi:10.1007/s10654-020-00640-5 32367290 PMC7250962

[23] Leening MJG , Kavousi M, Heeringa J, et al Methods of data collection and definitions of cardiac outcomes in the Rotterdam Study. Eur J Epidemiol. 2012;27:173–185. doi:10.1007/s10654-012-9668-8 22388767 PMC3319884

[24] WHO | Definition and diagnosis of diabetes mellitus and intermediate hyperglycaemia (Internet). WHO (cited May 12, 2021). Available from: https://www.who.int/publications/i/item/definition-and-diagnosis-of-diabetes-mellitus-and-intermediate-hyperglycaemia

[25] Inker LA , Schmid CH, Tighiouart H, et al.; CKD-EPI Investigators. Estimating glomerular filtration rate from serum creatinine and cystatin C. N Engl J Med. 2012; 367: 20–29. doi:10.1056/NEJMoa1114248 22762315 PMC4398023

[26] Burger H , de Laet CEDH, van Daele PLA, et al Risk factors for increased bone loss in an elderly population the Rotterdam Study. Am J Epidemiol. 1998;147:871–879. doi:10.1093/oxfordjournals.aje.a009541 9583718

[27] WHO | Haemoglobin concentrations for the diagnosis of anaemia and assessment of severity (Internet). WHO (cited May 12, 2021). Available from: https://apps.who.int/iris/handle/10665/85839

[28] DeLong ER , DeLong DM, Clarke-Pearson DL. Comparing the areas under two or more correlated receiver operating characteristic curves: a nonparametric approach. Biometrics. 1988;44: 837–845. doi:10.1111/aas.12728 3203132

[29] Lahousse L , Ziere G, Verlinden VJA, et al Risk of frailty in elderly with COPD: a population-based study. J Gerontol A Biol Sci Med Sci. 2016;71:689–695. doi:10.1093/gerona/glv154 26355016

[30] Huang F , Yang X, Yuan L, et al Development and validation of a predictive risk model for frailty in elderly patients with multimorbidity. Geriatr Gerontol Int. 2022;22:471–476. doi:10.1111/ggi.14390 35485599

[31] Varmaghani M , Dehghani M, Heidari E, Sharifi F, Moghaddam SS, Farzadfar F. Global prevalence of chronic obstructive pulmonary disease: systematic review and meta-analysis. East Mediterr Health J Rev. 2019;25:47–57. doi:10.26719/emhj.18.014 30919925

[32] Zhang D , Tang W, Dou L-Y, Luo J, Sun Y. Four different frailty models predict health outcomes in older patients with stable chronic obstructive pulmonary disease. BMC Geriatr. 2022;22:57. doi:10.1186/s12877-022-02750-z 35034605 PMC8761265

[33] Roberts MH , Mapel DW, Ganvir N, Dodd MA. Frailty among older individuals with and without COPD: a cohort study of prevalence and association with adverse outcomes. Int J Chron Obstruct Pulmon Dis Dove Press. 2022;17:701–717. doi:10.2147/COPD.S348714 PMC8994612 35411140

[34] Scarlata S , Finamore P, Laudisio A, et al Association between frailty index, lung function, and major clinical determinants in chronic obstructive pulmonary disease. Aging Clin Exp Res. 2021;33:2165–2173. doi:10.1007/s40520-021-01878-z 34009526

[35] Buhr RG , Barjaktarevic IZ, Quibrera PM, et al; SPIROMICS Investigators. Reversible airflow obstruction predicts future chronic obstructive pulmonary disease development in the SPIROMICS cohort: an observational cohort study. Am J Respir Crit Care Med. 2022;206:554–562. doi:10.1164/rccm.202201-0094OC 35549640 PMC9716898

