# Supplementary material for: Frailty Transitions in Older Persons With Lung Function Impairment: A Population-Based Study
Source: J Gerontol A Biol Sci Med Sci. 2022 Oct 13;78(2):349–56. doi: 10.1093/gerona/glac202 (PMC9951055; doi:10.1093/gerona/glac202)
Supplement: glac202_suppl_Supplementary_Material [file glac202_suppl_supplementary_material.pdf]

Frailty transitions in older persons with lung function impairment:

a population-based study

Sara R. A. Wijnant[1,2,3]; Elizabeth Benz[2,4], Annemarie I. Luik[2]; Fernando Rivadeneira[4], Trudy Voortman[2, 5] Guy G. Brusselle[1,2,6], Lies Lahousse[2,3]

[1]Department of Respiratory Medicine, Ghent University Hospital, Ghent, Belgium.
[2]Department of Epidemiology, Erasmus MC University Medical Center, Rotterdam, the Netherlands.
[3]Department of Bioanalysis, Faculty of Pharmaceutical Sciences, Ghent University, Ghent, Belgium.
[4]Department of Internal Medicine, Erasmus MC – University Medical Center Rotterdam, Rotterdam, the Netherlands.
[5]Division of Human Nutrition and Health, Wageningen University & Research, Wageningen, the Netherlands.
[6]Department of Respiratory Medicine, Erasmus MC – University Medical Center Rotterdam, Rotterdam, the Netherlands.

Correspondence:
Lies Lahousse, PhD
Department of Bioanalysis,
Faculty of Pharmaceutical Sciences, Ghent University, Ghent
Email: lies.lahousse@ugent.be

Supplementary files

Supplementary Figures

e-figure S1. Flowchart of participants

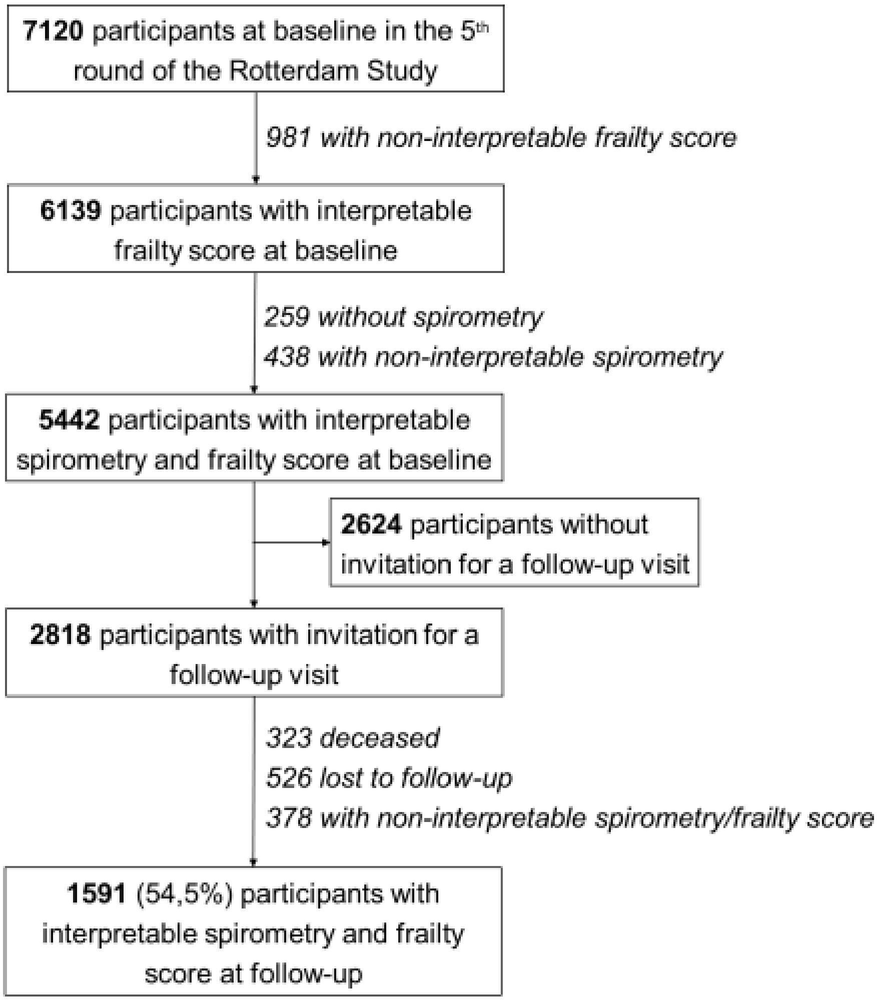

e-figure S2. Kaplan-Meier plot for survival in subjects with frailty and spirometry measures at baseline (n=5414)

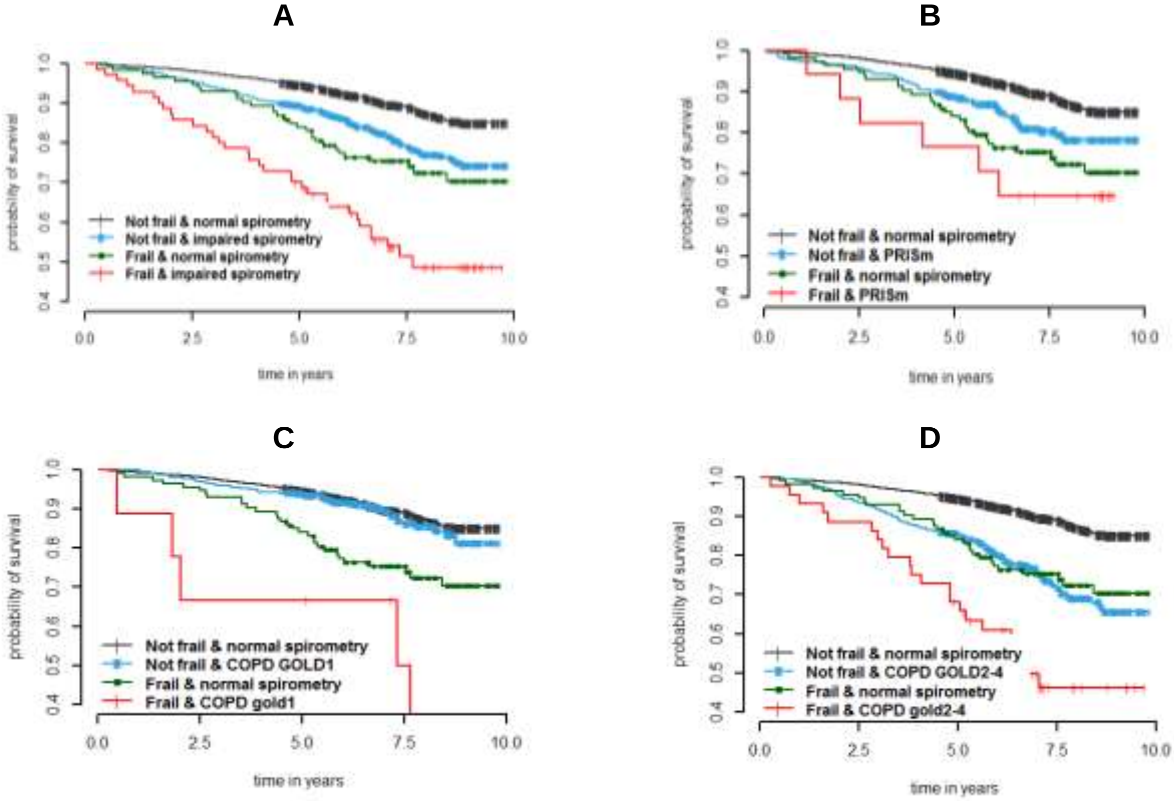

COPD = Chronic Obstructive Pulmonary Disease, GOLD = Global Initiative for Obstructive Lung Diseases, PRISm = Preserved Ratio Impaired Spirometry.

Supplementary Tables

e-table S1. Definition of frailty

Criteria	Instrument	Definition
Weight loss	Directly measured weight at each center visit	> 5% body weight lost compared with the previous examination
Low physical activity	Adapted version of the Zutphen Physical Activity Questionnaire [15]	Expending < 383 kcal/week for men and < 270 kcal/week for women
Slow gait velocity	5.79-m long walkway (GAITRite^{TM} Platinum; CIR systems, Sparta, NJ: 4.88-m active area; 120-Hz sampling rate)[16]	Walking at a velocity of < 0.76 m/s if height was > 173 cm for men or > 159 cm for women<br>Walking at a velocity of < 0.65 m/s if height was ≤ 173 cm for men or ≤ 159 cm for women
Reduced grip strength	Hydraulic hand dynamometer (Fabrication Enterprises Inc., White Plains, NY, USA)	≤ 29 kg grip strength if body mass index (BMI) ≤ 24, ≤ 30 kg grip strength if BMI 24.1-28 or ≤ 32 kg grip strength if BMI > 28 for men<br>≤ 17 kg grip strength if BMI ≤ 23, ≤ 17.3 kg grip strength if BMI 23.1-26, ≤ 18 kg grip strength if BMI 26.1-29, or ≤ 21 kg grip strength if BMI > 29 for women
Exhaustion	Dutch translation of the Center for Epidemiological Studies Depression Scale [17, 18]	Answering "frequently" or "mostly" to one of the following two statements: (a) I felt that everything I did was an effort; (b) I could not get going.

e-table S2. Baseline characteristics of participants that meet the criteria for PRISm or COPD (n=5442)

	Normal spirometry N=4158	PRISm N=381	P*	COPD N=903	P*
Age (years)	68.8 (8.7)	69.7 (9.7)	0.051	**70.5 (9.1)**	**<0.001**
Female sex (%)	2415 (58.1%)	221 (58.0%)	1.000	**402 (44.5)**	**<0.001**
BMI (kg/m²)	27.6 (4.3)	**29.5 (5.1)**	**<0.001**	**26.5 (4.0)**	**<0.001**
Current smoking (%)	377 (9.1%)	**54 (14.2%)**	**0.002**	**233 (25.8)**	**<0.001**
Packyears (years)	3.0 (0.0-18.6)	**8.3 (0.0-27.0)**	**0.006**	**21.0 (3.0-41.3)**	**<0.001**
Total cholesterol (mmol/l)	5.5 (1.1)	**5.3 (1.2)**	**<0.001**	**5.3 (1.0)**	**<0.001**
Glucose (mmol/l)	5.8 (1.3)	**6.1 (1.4)**	**<0.001**	5.8 (1.2)	0.954
Hemoglobin (mmol/l)	8.8 (0.7)	**8.7 (0.8)**	**0.008**	**8.9 (0.8)**	**<0.001**
White blood cell count (#,*10^3)	6.9 (1.9)	**7.5 (2.0)**	**<0.001**	**7.6 (2.4)**	**<0.001**
Serum creatinine (µmol/l)	80.1 (20.7)	80.0 (24.3)	0.953	83.2 (25.0)	<0.001
FEV_1 % predicted	104.0 (13.2)	**71.8 (7.2)**	**<0.001**	**77.1 (18.2)**	**<0.001**
FVC % predicted	101.7 (12.9)	**72.0 (8.6)**	**<0.001**	**92.5 (17.6)**	**<0.001**
FEV_1/FVC (%)	78.8 (4.7)	**76.9 (5.1)**	**<0.001**	**63.3 (6.6)**	**<0.001**

BMI = Body Mass Index, COPD = Chronic Obstructive Pulmonary Disease, FEV_1 = Forced Expiratory Volume in one second, FVC = Forced Vital Capacity, PRISm = Preserved Ratio Impaired Spirometry. *P values are comparing characteristics of participants with PRISm or COPD, respectively, with participants that have normal spirometry.

e-table S3. Baseline characteristics of participants with and without re-examination

	Without re-examination N=1227	With re-examination N=1591	P
Age (years)	**77.9 (6.1)**	**73.8 (4.98)**	**<0.001**
Female sex (%)	698 (56.9)	875 (55.0)	0.335
BMI (kg/m²)	27.5 (4.2)	27.5 (4.1)	0.998
Current smoking (%)	**148 (12.1)**	**116 (7.3)**	**<0.001**
Packyears (years)	**7.2 (0.0-27.8)**	**4.9 (0.0-22.0)**	**0.015**
Total cholesterol	**5.3 (1.1)**	**5.4 (1.1)**	**<0.001**
Glucose	5.8 (1.2)	5.8 (1.2)	0.235
Hemoglobin	**8.6 (0.8)**	**8.8 (0.7)**	**<0.001**
White blood cell count	**7.3 (2.2)**	**6.9 (1.7)**	**<0.001**
Serum creatinine	**84.4 (29.6)**	**80.8 (19.7)**	**<0.001**
Prefrailty (%)	**774 (63.1)**	**859 (54.0)**	**<0.001**
Frailty (%)	**105 (8.6)**	**49 (3.1)**	**<0.001**
PRISm (%)	**117 (9.5)**	**87 (5.56)**	**<0.001**
COPD (%)	**270 (22.0)**	**236 (14.9)**	**<0.001**
FEV_1 % predicted	**94.1 (20.9)**	**99.0 (18.3)**	**<0.001**
FVC % predicted	**94.8 (17.4)**	**99.0 (15.3)**	**<0.001**
FEV_1/FVC	**75.0 (8.6)**	**76.2 (7.4)**	**<0.001**

BMI = Body Mass Index, COPD = Chronic Obstructive Pulmonary Disease, FEV_1 = Forced Expiratory Volume in one second, FVC = Forced Vital Capacity, PRISm = Preserved Ratio Impaired Spirometry.

e-table S4. Baseline characteristics by frailty transition state

	Stable robust	Recovery	Progression	Stable frail
	Stable robust[A] N=297	Prefrail/frail to robust[B] N=157	Robust to prefrail/frail[A] N=386	Stable prefrail/frail[B] N=751
Age (years)	**71.9 (3.8)**	**72.4 (4.2)**	**73.4 (4.4)**	**75.1 (5.4)**
Female sex (%)	138 (46.5%)	90 (57.3%)	189 (49.0%)	458 (39.0%)
BMI (kg/m^2)	**26.3 (3.3)**	**26.6 (3.8)**	**27.3 (3.6)**	**28.3 (4.4)**
Current smoking (%)	24 (8.1%)	17 (10.8%)	20 (5.2%)	55 (7.3%)
Packyears (years)	4.5 (0.0-21.6)	2.4 (0.0-25.0)	3.7 (0.0-21.0)	6.0 (0.0-23.0)
Total cholesterol	5.6 (1.1)	5.4 (1.1)	5.5 (1.1)	5.4 (1.1)
Glucose	**5.5 (0.8)**	5.7 (1.5)	**5.8 (1.2)**	5.9 (1.3)
Hemoglobin	8.9 (0.6)	**8.8 (0.7)**	8.9 (0.8)	**8.7 (0.8)**
White blood cell count	6.7 (1.6)	7.0 (1.8)	6.9 (1.7)	7.0 (1.7)
Serum creatinine	81.8 (16.1)	80.7 (23.7)	81.8 (17.8)	79.8 (20.9)
Slow gait speed (%)	0 (0.0)	2 (1.8)	0 (0.0)	10 (2.1)
Δ gait speed rate	-1.5 (3.1)	-1.5 (2.3)	-2.1 (3.4)	-2.3 (3.1)
Weight loss (%)	0 (0.0)	**89 (58.2)**	0 (0.0)	**202 (27.6)**
Δ weight rate	-0.0 (0.5)	0.1 (0.8)	-0.4 (0.9)	-0.3 (1.0)
Low physical activity (%)	0 (0.0)	17 (11.7)	0 (0.0)	125 (18.5)
Δ physical activity rate	-164.1 (131.7)	-134.4 (156.4)	-144.6 (131.6)	-110.8 (138.3)
Low grip strength (%)	0 (0.0)	**51 (32.9)**	0 (0.0)	**534 (71.7)**
Δ grip strength rate	-0.5 (0.9)	0.3 (1.2)	-1.2 (1.1)	-0.5 (1.0)
Exhaustion (%)	0 (0.0)	29 (18.6)	0 (0.0)	171 (22.8)
PRISm (%)	10 (3.4%)	10 (6.3%)	19 (4.9%)	48 (6.4%)
COPD (%)	46 (15.5%)	15 (9.5%)	47 (12.2%)	48 (6.4%)
FEV % predicted	101.2 (16.6)	**101.3 (18.4)**	100.8 (18.3)	**96.9 (18.7)**
FEV1 decline rate (ml/year)	-42.5 (50.9)	-47.8 (51.2-	-40.9 (57.5)	-44.6 (51.7)
FVC % predicted	101.0 (14.0)	**101.4 (16.6)**	100.4 (15.0)	**96.9 (15.4)**
FVC decline rate (ml/year)	-10.0 (73.3)	-18.4 (69.0)	-10.1 (75.5)	-19.2 (69.1)
FEV_1/FVC	76.4 (6.7)	76.4 (6.2)	76.3 (7.1)	76.1 (8.0)
Deaths (%)	8 (2.7)	7 (4.5)	12 (3.1)	68 (9.1)

BMI = Body Mass Index, COPD = Chronic Obstructive Pulmonary Disease, FEV_1 = Forced Expiratory Volume in one second, FVC = Forced Vital Capacity, PRISm = Preserved Ratio Impaired Spirometry. [A]P values (**p<0.05**) are comparing characteristics of robust participants at baseline between those that rmain robust and those that progress towards prefrailty/frailty. [B]P values (**p<0.05**) are comparing characteristics of prefrail/frail participants at baseline between those that remain prefrail/frail and those that recover towards robust.

e-table S5. Estimates of Mortality in the total population

Group	At risk (#)	Deaths (#)	CI (%)	IR (#/1000 PY)	IRR	aHR (95% CI)
Total	5851	804	13.7	21.0	NA	NA
Spirometry (5414)						
Normal spirometry	4135	425	10.3	15.5	Ref	Ref
PRISm	379	68	17.9	28.2	1.8	**1.6 (1.3,2.1), p<0.001**
COPD	900	185	20.6	32.2	2.1	**1.6 (1.4,1.9), p<0.001**
Frailty (n=5414)						
Robust	2590	157	6.1	9.4	Ref	Ref
Prefrail	2641	458	17.3	26.1	2.8	**1.7 (1.4,2.1), p<0.001**
Frail	183	63	34.4	52.7	5.6	**2.4 (1.8,3.3), p<0.001**
Frailty and lung function (n=5414)						
Not frail & normal spirometry	4022	395	9.8	14.8	NP	NP
Not frail but PRISm or COPD	1209	220	18.2	28.4	NP	NP
Frail & normal spirometry	113	30	26.5	38.3	Ref	Ref
Frail & PRISm	17	6	35.3	52.0	1.4	1.4 (0.6,3.4), p=0.431
Frail & COPD	53	27	50.9	90.6	**2.4**	**1.9 (1.1,3.1), p=0.020**

HR = Hazard Ratio; PRISm = Preserved Ratio Impaired Spirometry; COPD = Chronic Obstructive Pulmonary Disease; PY = person-years; NA = not applicable; NP = Not Performed; aHR = HR adjusted for age and sex.

e-table S6. Comparison of diagnostic accuracy for predicting mortality in subjects with COPD (n=900)

	1-year mortality	3-year mortality	5-year mortality	9-year mortality
	AUC (95% CI)	AUC (95% CI)	AUC (95% CI)	AUC (95% CI)
Model 1				
Age, sex, smoke	77.9 (67.2-88.6)	75.3 (69.1-81.4)	77.8 (73.1-82.5)	82.4 (79.1-85.7)
+ FEV1 % pred	85.5 (76.1-94.9)	77.6 (72.2-83.0)	**80.9 (76.9-85.0)**	<u>**85.0 (82.1-87.9)**</u>
+ FVC % pred	84.2 (74.6-93.8)	77.3 (71.8-82.8)	**80.2 (76.1-84.3)**	<u>**84.3 (81.3-87.8)**</u>
+ frailty score	**90.5 (82.3-98.8)**	**78.9 (73.7-84.2)**	**79.6 (75.4-83.9)**	**83.4 (80.2-86.5)**
+ dyspnea score	88.0 (80.3-95.8)	75.5 (68.8-82.2)	78.9 (74.1-83.7)	83.1 (79.7-86.5)
+ comorbidity score	84.0 (69.8-98.2)	77.6 (71.5-83.7)	<u>**80.8 (76.5-85.1)**</u>	<u>**84.2 (81.0-87.3)**</u>
Model 2				
Age, sex, smoke, frailty	90.5 (82.3-98.8)	78.9 (73.7-84.2)		
+ FEV1 % pred	90.3 (80.6-100.0)	80.1 (75.1-85.0)		
+ FVC % pred	89.7 (78.9-100.0)	79.9 (74.9-84.9)		
+ comorbidity score	92.6 (86.5-98.8)	80.1 (74.9-75.4)		
Age, sex, smoke, FEV1 %			80.9 (76.9-85.0)	85.0 (82.1-87.9)
+ frailty score			81.9 (78.0-85.7)	85.5 (82.7-88.3)
+ comorbidity score			**83.0 (79.1-86.8)**	**86.1 (83.3-88.9)**
Model 3				
Age, sex, smoke, FEV1 %, comorbidity score			83.0 (79.1-86.8)	86.1 (83.3-88.9)
+ frailty score			83.6 (79.9-87.2)	86.5 (83.8-89.3)

AUC's (95% CI) for predicting mortality in subjects with COPD are shown. Model 1 is adjusted for age, sex and smoking history, added with either FEV_1 % predicted, FVC % predicted, frailty score, dyspnea score or comorbidity score. Model 2 and 3 are adjusted for variables in model 1 and 2, respectively, as well as the additional covariable from model 1 or 2, respectively, yielding the largest AUC, consequently added with the remaining covariables. FEV_1 = Forced Expiratory Volume in one second, FVC = Forced Vital Capacity. P values of Chi-square tests from the nonparametric method described by DeLong et al. are presented to compare differences in AUC. <u>**P<0.01**</u> **is indicated underlined and bold, p<0.05 is indicated bold.**
